# Folic Acid Mitigates Sertraline-Induced Liver Damage in Adult Female Albino Rats During Pregnancy and Postpartum: A Biochemical and Histological Study

**DOI:** 10.3390/medicina61040751

**Published:** 2025-04-18

**Authors:** Ayman A. Refai, Mohammad I. Jumaa, Einas M. Yousef, Ala M. Aljehani, Rana Ahmed Alduraywish, Mohamed R. Elkabary, Safaa M. Hanafy, Hanan S. Seleem, Eman S. El-Roghy

**Affiliations:** 1 Department of Anatomy and Physiology, College of Medicine, Imam Mohammad Ibn Saud Islamic University (IMSIU), Riyadh 13317, Saudi Arabia; aarefai@imamu.edu.sa (A.A.R.);; 2 College of Medicine, Alfaisal University, Riyadh 11533, Saudi Arabia; 3 Department of Histology and Cell Biology, Faculty of Medicine, Menoufia University, Shebin el Kom 32511, Egypte.shehata27@yahoo.com (E.S.E.-R.); 4 Department of Pathology, College of Medicine, Imam Mohammad Ibn Saud Islamic University (IMSIU), Riyadh 13317, Saudi Arabia; 5 Nutrition and Food Science Department, Faculty of Home Economic, Menoufia University, Shebin el Kom 32511, Egypt; m_elkabary@yahoo.com; 6 Anatomy Department, Faculty of Medicine (Girls), Al-Azhar University, Cairo 11754, Egypt; 7 Department of Anatomy, College of Medicine, Jouf University, Skaka 72388, Saudi Arabia

**Keywords:** apoptosis, caspase 3, folic acid, hepatotoxicity, pregnancy, sertraline

## Abstract

*Background and Objectives:* Sertraline is a selective serotonin reuptake inhibitor (SSRI) that is frequently prescribed during pregnancy to treat mood disorders. Studies indicate that chronic use of sertraline is associated with elevated liver enzymes, oxidative stress, and histological alterations in the liver. Folic acid, a recommended supplement currently used during the first trimester of pregnancy, has antioxidant and anti-inflammatory effects. Hence, folic acid might be a potential protective agent against sertraline-induced liver injury. The current study aimed to investigate the possible hepatotoxic effects of sertraline administration during pregnancy and early postpartum. In addition, we sought to evaluate the potential protective effects of folic acid supplementation in alleviating any sertraline-induced liver damage. *Materials and Methods:* Eighty pregnant albino rats were randomly divided into four groups: control, folic acid-treated, sertraline-treated, and folic acid–sertraline-treated. Each group was divided into rats euthanized immediately after giving birth (0 h) or 14 days postpartum. Biochemical, histological, and immunohistochemical evaluations of liver function and structure were conducted. *Results:* Administration of sertraline was associated with a significant increase in hepatic enzymes (ALT and AST) and disrupted lipid profile (elevated cholesterol, triglycerides, and LDL-c) compared to the control group. Increased apoptosis was evidenced by increased caspase 3 expression and histological alterations, including vacuolation and inflammatory infiltrates, in sertraline-treated rats. Folic acid supplementation effectively mitigated these effects by preserving liver architecture, normalizing biochemical markers (ALT, AST, and lipid profile changes), and reducing apoptotic activity (lower caspase 3 expression). *Conclusions:* Folic acid mitigated sertraline-induced hepatic damage in pregnant rats. This suggests the potential benefits of using folic acid during the whole duration of pregnancy in patients treated with sertraline.

## 1. Introduction

Pregnancy is a process that involves extensive physiological and hormonal changes that affect women’s organs and tissues. This period is critical not only for mothers’ health but also for the growth and development of babies [1]. Pregnant women are more susceptible to mood disorders such as antenatal depression, which is characterized by sadness, anxiety, fatigue, and sleep disturbance [2]. These conditions may require the administration of some antidepressants, such as selective serotonin reuptake inhibitors (SSRIs), to support maternal mental health during pregnancy.

Sertraline, a well-known SSRI, is one of the most prescribed antidepressants during pregnancy for the management of depression, anxiety, and other mood-related disorders [3]. It increases serotonin levels in the brain, which helps to alleviate symptoms of mood disorders [4]. Sertraline is metabolized in the liver primarily through the cytochrome P450 enzyme system (CYP2C19, CYP2B6, and CYP3A4). While sertraline is considered to have low potential for hepatotoxicity, recent studies have demonstrated that it can cause alterations in liver enzymes in animal studies, indicating potential hepatic stress [5,6]. These alterations include abnormal liver enzymes such as ALT, AST, and ALP, as well as abnormal bilirubin levels after chronic administration, which might indicate hepatocellular damage or cholestasis. Moreover, sertraline’s effects on the liver during pregnancy could be further complicated by hormonal changes, such as increased estrogen and progesterone levels, which can influence drug metabolism and liver enzyme activity [7].

Folic acid, a water-soluble vitamin (B9), is widely recognized as an essential nutrient that is widely recommended during the first trimester of pregnancy. It contributes to the overall health of pregnant women, supports metabolic functions, and is crucial for preventing neural tube defects. It plays a key role in DNA synthesis, cell division, cellular and tissue growth, and regeneration, which are essential for maternal health during pregnancy and fetal development. Incorporating folic acid-rich foods, such as green leaves and citrus fruits, in the diet, in addition to supplementations during pregnancy, can significantly support liver health [8]. Folic acid has antioxidant and anti-inflammatory properties that may be beneficial in protecting liver cells from oxidative stress and drug-induced liver damage [9]. It has been reported that folic acid can modulate the body’s response to toxins and drugs by reducing homocysteine levels and increasing glutathione synthesis. This powerful antioxidant protects liver cells from damage [10].

Folic acid has been shown to prevent changes in liver enzyme levels and histopathological alterations in liver tissue [11]. Previous studies demonstrated that folic acid deficiency is associated with protein regulation disruptions, increased liver proinflammatory factors, and impaired lipid metabolism, resulting in excess fat accumulation in hepatocytes and liver fibrosis [2,12,13]. Histopathological examination in previous studies has further revealed that the accumulation of hepatic lipids and inflammatory infiltration is recognized as a hallmark of liver diseases [12]. Furthermore, low folic acid levels have been correlated with the pathogenesis of liver diseases, such as hepatitis C, chronic hepatitis, liver cirrhosis, and hepatic carcinoma [14].

Although sertraline is generally considered safe during pregnancy, it has potential hepatotoxic effects, including changes in liver enzyme levels or—in rare cases—severe liver damage [15]. Folic acid, commonly prescribed during the first trimester of pregnancy, may help mitigate liver damage caused by sertraline by supporting liver function, reducing oxidative stress, and promoting cellular repair. Moreover, folic acid has been shown to play a synergistic role when administered with SSRIs by enhancing antidepressant efficacy and improving treatment outcomes. For example, Coppen and Bailey (2000) reported that folate supplementation augmented the therapeutic effects of fluoxetine in depressed patients [16]. Such findings provided additional support for exploring the combination of folic acid with sertraline in our experimental design. The current study aimed to evaluate the potential hepatotoxic effects of sertraline in pregnancy and postpartum period of female albino rats and to investigate the possible protective role of folic acid supplementation. By exploring this interaction, we sought to provide insights into safer pharmacological practices during pregnancy, particularly for individuals with SSRI prescriptions.

## 2. Materials and Methods

### 2.1. Drugs

Sertraline tablets were purchased from Pfizer, a local company in Cairo, Egypt. The folic acid was bought from Nile Pharmaceutical Company, Shibin El-Kom City, Menoufia Governorate, Egypt. Sertraline (Zoloft) is a well-known antidepressant drug with a recommended human dose of 50 mg/day administered orally [17]. Based on this, the dose of sertraline for an adult rat (weighing 200 g) was calculated to be 0.9 mg/rat/day based on the recommendations of Paget and Barnes [18]. Accordingly, 0.9 mg sertraline was prepared in 0.5 mL of distilled water for each rat. This was achieved by dissolving one sertraline tablet in 27.75 mL of distilled water.

The guidelines of Paget and Barnes [18] were also followed to prepare folic acid. The recommended folic acid dose for a pregnant human patient receiving antidepressant drugs is 4 mg/day [19]. Hence, the equivalent dose for a 200 g adult rat was calculated to be 0.072 mg/rat/day, to be administered orally. To achieve this dose, each folic acid tablet was dissolved in 40.3 mL of distilled water, and each rat was gavaged with 0.58 mL of the solution daily.

### 2.2. Animals

#### 2.2.1. Animals Used in the Experiment

Eighty pregnant Wistar female albino rats aged 3–4 months were used in this study. Rats within this age range are considered young adults, representing a stage of full physiological and behavioral maturity [20]. This stage ensures stable metabolic, endocrine, and behavioral profiles, which are critical for consistent pharmacological and toxicological assessments [21]. Female rats at this age are also reproductively mature, providing a reliable model for studies involving hormonal status and postpartum physiology while avoiding variability associated with immature or aged reproductive cycles. Albino strains such as Wistar are commonly used due to their well-documented genetic backgrounds and standardized responses across a wide range of experimental paradigms, further enhancing reproducibility [22]. The selection of this model was also supported by its relevance to translational research, as the physiological characteristics of young adult rats approximate several aspects of human physiology [23].

During the experimental period, the rats were kept in an animal house under standardized environmental conditions (21 ± 2 °C, 50% ± 10% relative humidity, and 12 h light/dark cycle). The experiment was carried out in the animal house at the Faculty of Medicine, Menoufia University. The study was conducted after acquiring ethics approval from the Institutional Animal Care and Use Committee of Menoufia University, Egypt (approval MUFHE/F/NFS/6/25).

#### 2.2.2. Experimental Design

**Figure 1:**
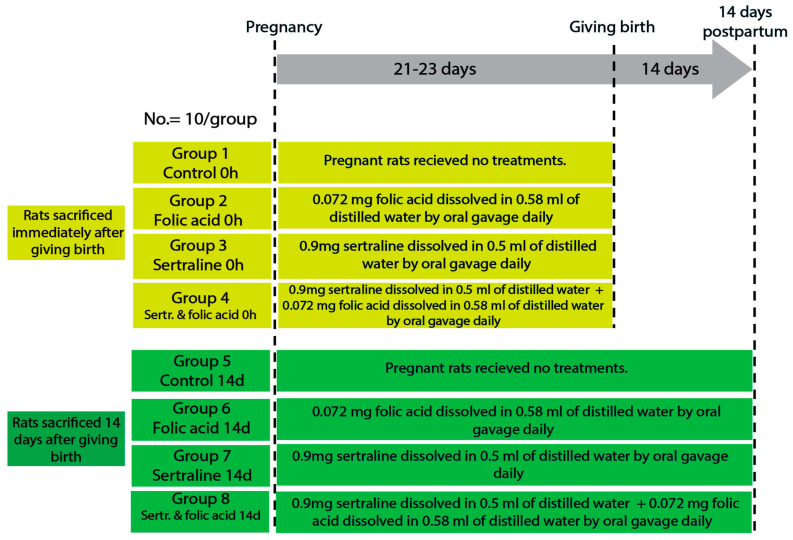
Diagram showing the experimental design, animal groups, and timeline.

Female rats were housed with males for mating, and pregnancy was confirmed by the presence of a vaginal plug, which was considered pregnancy day zero. The gestation period of rats is approximately 21–23 days, divided into early (0–7 days), mid (8–14 days), and late (15–21 days) pregnancy [24]. The pregnant rats were randomly divided into four main groups: control, folic acid-treated, sertraline-treated, and folic acid–sertraline-treated. Then, each group was subdivided into two subgroups based on the time of death (0 h after giving birth and 14 days postpartum; 10 per subgroup) (Figure 1).

The sample size (10 rats per group) was determined based on guidelines for animal research design described by Charan and Kantharia (2013), which recommend a minimum of 6–10 animals per group for detecting moderate effect sizes in preclinical studies [25]. Moreover, the chosen sample size has been widely adopted in similar experimental models and is generally considered sufficient for detecting statistically and biologically relevant effects in biochemical and histological assessments.

The groups were as follows.

- Group 1 (control 0 h): pregnant rats were fed a balanced diet throughout the pregnancy and euthanized immediately after giving birth.
- Group 2 (folic acid 0 h): pregnant rats were fed a balanced diet and administered 0.072 mg folic acid dissolved in 0.58 mL of distilled water by oral gavage daily throughout the pregnancy, then euthanized immediately after giving birth.
- Group 3 (sertraline 0 h): pregnant rats were fed a balanced diet and administered 0.9 mg sertraline dissolved in 0.5 mL of distilled water by oral gavage daily throughout the pregnancy, then euthanized immediately after giving birth.
- Group 4 (folic acid + sertraline 0 h): pregnant rats were fed a balanced diet and administered 0.072 mg folic acid dissolved in 0.58 mL of distilled water, followed by administering 0.9 mg sertraline dissolved in 0.5 mL of distilled water by oral gavage daily throughout the pregnancy, then euthanized immediately after giving birth.
- Group 5 (control 14 d): pregnant rats were fed a balanced diet throughout the pregnancy and for 14 days after giving birth.
- Group 6 (folic acid 14 d): pregnant rats were fed a balanced diet and administered 0.072 mg folic acid dissolved in 0.58 mL of distilled water by oral gavage daily throughout the pregnancy and for 14 days postpartum.
- Group 7 (sertraline 14 d): pregnant rats were fed a balanced diet and administered 0.9 mg sertraline dissolved in 0.5 mL of distilled water by oral gavage daily throughout the pregnancy and for 14 days postpartum.
- Group 8 (folic acid + sertraline 14 d): pregnant rats were fed a balanced diet and administered 0.072 mg folic acid dissolved in 0.58 mL of distilled water, followed by administration 0.9 mg sertraline dissolved in 0.5 mL of distilled water by oral gavage daily throughout the pregnancy and for 14 days postpartum.

### 2.3. Blood and Tissue Sample Collection

By the end of the experiment, rats had been euthanized at two time points based on their groups. For groups 1, 2, 3, and 4, animals were euthanized immediately after giving birth. For groups 5, 6, 7, and 8, animals were euthanized 14 days postpartum. At each time point, rats were anesthetized by intraperitoneal injection of 60 mg/kg ketamine and 5 mg/kg xylazine. After that, all animals were euthanized via decapitation. Blood samples were collected from the abdominal aorta and kept at room temperature for 30 min. The samples were centrifuged at 1200 relative centrifugal force (rcf) for 10 min to collect the carefully aspirated serum and transferred into clean cuvette tubes, then kept at −20 °C for further biochemical analysis. Livers were excised, sectioned, and fixed in formalin 10% for further processing for histological examination.

### 2.4. Biochemical Analysis

#### 2.4.1. Lipid Profile

Lipid profiles were analyzed following the analytical procedure described by Khalil et al. [26]. Serum total cholesterol (CHO) was measured according to methods described in [27]. Serum triglycerides (TGs) were assessed by the enzymatic method described by Fossati and Prencipe [28] using commercially available kits. Moreover, high-density-lipoprotein cholesterol (HDL-c) was measured using the method described in [29,30]. Finally, low-density-lipoprotein cholesterol (LDL-c) was calculated (mg/dL) using the equation LDL-c (mg/dL) = Total CHO − (HDL-c + vLDL-c), as described by Okada et al. [31].

#### 2.4.2. Liver Function

As per Kaplan et al. [32], hepatic functions were evaluated by measuring serum alanine aminotransferase (ALT) and aspartate aminotransferase (AST) levels. All methods were applied with slight modifications, as detailed in recently published research by Aljumayi et al. [33].

### 2.5. Histological Analysis

Specimens of the liver were immediately fixed in 10% neutral-buffered formalin, dehydrated, cleared, and then embedded in paraffin. Sections of 5 μm thickness were then cut using a microtome and mounted onto glass slides. The sections were stained with hematoxylin and eosin (H&E) for general histological examination under a light microscope. Histological changes were assessed semiquantitatively using the scoring system described by Suzuki et al., evaluating three parameters: sinusoidal congestion, cytoplasmic vacuolization of hepatocytes, and parenchymal necrosis. Each parameter was scored on a scale from 0 to 4, where 0 indicates no pathological changes, 1 indicates minimal changes, 2 indicates mild changes affecting up to 30% of the tissue, 3 indicates moderate changes involving up to 60%, and 4 indicates severe damage affecting more than 60% [34].

### 2.6. Immunohistochemical Analysis

Liver sections (4–5 µm in thickness) from all experimental groups were subjected to immunohistochemical staining for caspase 3, a key apoptosis marker. The staining was carried out using a rabbit monoclonal antibody (clone 9H19L2, catalogue number 700182) obtained from Lab Vision Corporation, USA. After deparaffinization and rehydration, antigen retrieval was performed by heating the slides in a citrate buffer (pH 6.0) in a microwave for 10 min. The slides were incubated with 3% hydrogen peroxide for 10 min to block endogenous peroxidase activity, followed by a blocking buffer (e.g., 5% bovine serum albumin) for 30 min at room temperature. The sections were incubated overnight at 4 °C with primary antibodies specific to caspase 3. The slides were washed with phosphate-buffered saline (PBS), incubated with a biotinylated secondary antibody, and then streptavidin–horseradish peroxidase (HRP) was applied for 30 min. The signal was visualized using a diaminobenzidine (DAB) chromogen substrate. Sections were then counterstained with hematoxylin, dehydrated, and mounted with a coverslip. The positive control used was mouse appendix, while omission of the primary antibody served as the negative control. Finally, morphometric analysis was conducted to assess the immunohistochemical staining of caspase 3 using an open-source IHC profiler plugin of ImageJ (Version 15.4f) on a random six field/slide by power field (×400) [35].

### 2.7. Statistical Analysis

The Statistical Package for Social Science (SPSS) version 22.0 (SPSS Inc., Chicago, IL, USA) was used for statistical analysis. Data are reported as means ± standard deviation (SD) for all study groups. Prior to conducting parametric tests, data were assessed for normality using the Shapiro–Wilk test and for homogeneity of variance using Levene’s test. Differences between groups were evaluated using one-way analysis of variance (ANOVA), followed by Tukey’s post hoc test for pairwise comparisons. A *p*-value < 0.05 was considered statistically significant.

## 3. Results

### 3.1. Lipid Profile Levels at Two Time Points Across Different Experimental Groups

Table 1 lists the data for the lipid profile parameters CHO, TGs, HDL-c, and LDL-c across experimental groups assessed immediately after giving birth and 14 days postpartum. At the first time point (immediately after giving birth), statistical analysis of mean total cholesterol, triacylglycerol, and low-density-lipoprotein levels in groups 1, 2, 3, and 4 revealed that groups 3 (sertraline 0 h) and 4 (sertraline and folic acid 0 h) had significantly higher levels than groups 1 (control 0 h) and 2 (folic acid 0 h). On the other hand, it showed a significant decline in group 4 (sertraline–folic acid-treated) relative to group 3 (sertraline-treated). Statistical analysis of the mean high-density lipoprotein levels in groups 1, 2, 3, and 4 revealed insignificant differences between the groups (Table 1).

At 14 days after giving birth, statistical analysis of mean total cholesterol, triacylglycerol, and low-density-lipoprotein levels in groups 5, 6, 7, and 8 revealed that groups 7 (sertraline 14 d) and 8 (sertraline and folic acid 14 d) had significantly higher levels than groups 3 (sertraline 0 h), 5 (control 14 d), and 6 (folic acid 14 d). On the other hand, it showed a significant decline in group 8 (sertraline and folic acid 14 d) relative to group 7 (sertraline 14 d). A statistical analysis of the mean high-density-lipoprotein levels in groups 5, 6, 7, and 8 revealed insignificant differences between the groups (Table 1).

### 3.2. Liver Functions at Two Time Points Across Different Experimental Groups

AST and ALT were assessed to evaluate the liver function status in all study groups (Table 2). At the first time point (immediately after giving birth), statistical analysis of the mean ALT and AST in groups 1, 2, 3, and 4 revealed that groups 3 (sertraline 0 h) and 4 (sertraline and folic acid 0 h) had significantly higher levels than groups 1 (control 0 h) and 2 (folic acid 0 h). On the other hand, it showed a significant decline in group 4 (sertraline and folic acid treated) relative to group 3 (sertraline treated) (Table 2).

After 14 days of giving birth, statistical analysis of the mean ALT and AST levels in groups 5, 6,7, and 8 revealed that groups 7 (sertraline 14 d) and 8 (sertraline and folic acid 14 d) had significantly higher levels than groups 3 (sertraline 0 h), 5 (control 14 d) and 6 (folic acid 14 d). On the other hand, it showed a significant decline in group 8 (sertraline and folic acid 14 d) relative to group 7 (sertraline 14 d) (Table 2).

### 3.3. Histological Analysis

**Figure 2:**
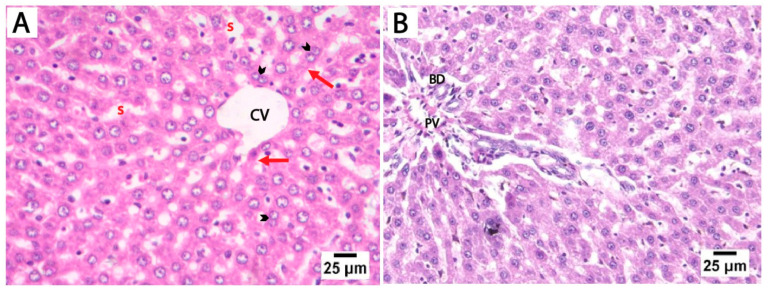
Photomicrographs of H&E-stained liver sections from control rats euthanized at two different time points. (**A**) Liver sections showing cords of hepatocytes that radiate outwards from the central vein (CV), separated by hepatic blood sinusoids (s). The hepatocytes (red arrows) appear as polyhedral cells with acidophilic cytoplasm and prominent vesicular nuclei, with some showing binucleation (arrowheads). (**B**) The portal tract of the control group showing connective tissue and a terminal branch of the portal vein (PV) and bile duct (BD) (scale bar 25 μm).

The H&E-stained liver sections from the control and folic acid groups from rats euthanized at two different time points displayed a normal microscopic structure of liver tissue, so they were pooled together. The hepatocytes appeared as polyhedral cells with acidophilic cytoplasm and prominent vesicular nuclei, some of which were binucleated. These cells were organized into cords radiating outward from the central vein, separated by hepatic sinusoids lined by flattened endothelial cells. The portal tracts at the periphery of the lobules contained connective tissue and terminal branches of the portal vein, hepatic artery, and bile duct (Figure 2).

**Figure 3:**
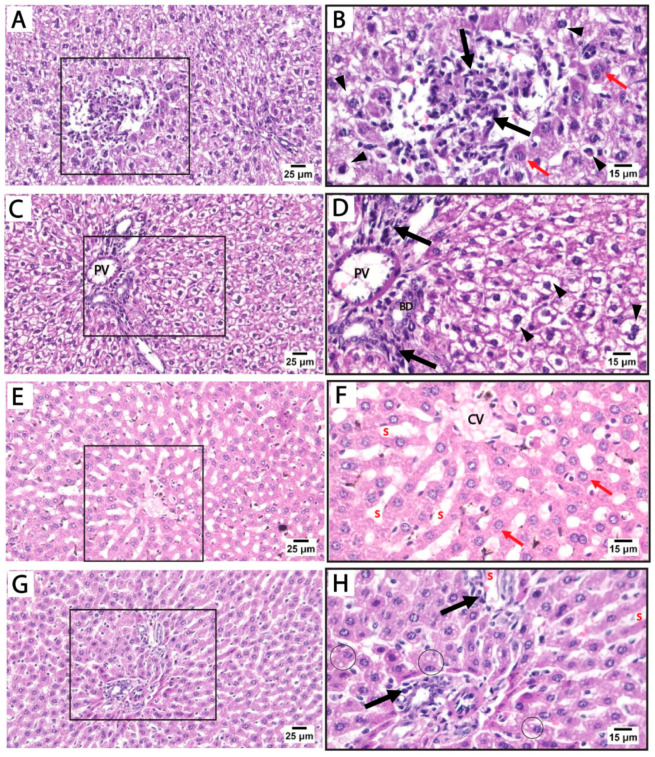
Photomicrographs of H&E-stained liver sections from treated rats that were euthanized immediately after giving birth. (**A**–**D**) treated with sertraline: (**A**,**B**) liver section showing inflammatory infiltrate (black arrow) of the hepatic parenchyma. Some hepatocytes appear normal (red arrows) with intact cytoplasm and vesicular nuclei. Some hepatocytes exhibit vacuolated cytoplasm with dark pyknotic nuclei (arrowheads). (**C**,**D**): Portal tract with a mildly dilated portal vein (PV) and intact bile duct (BD) with inflammatory infiltrate (black arrow). Disorganized cords of highly vacuolated, apparently hypertrophied hepatocytes (arrowheads) are detected near the portal tract. (**E**–**H**) treated with folic acid + sertraline: (**E**,**F**) Liver section displays relatively preserved hepatic architecture with organized cords of hepatocytes around distinct central veins (CV). The hepatocytes (red arrows) appear mostly normal, with intact cytoplasm and vesicular nuclei. (**G,H**) Liver section displays a portal area with minimal inflammatory infiltrate (black arrows) and relatively normal hepatic sinusoids (s). Slight cytoplasmic vacuolization (circles) is observed in a few hepatocytes. ((**A**,**C**,**E**,**G**): scale bar 25 μm), ((**B**,**D**,**F**,**H**): scale bar 15 μm), (**B**,**D**,**F**,**H**) are higher magnification of the areas marked by the square in (**A**,**C**,**E**,**G**), respectively.

Histological findings in pregnant rats immediately after giving birth: Light microscopy of H&E-stained liver sections from rats treated with sertraline and euthanized immediately after giving birth revealed a noticeable disorganization of the hepatic lobular pattern, as the hepatocyte cords are less distinct compared to normal hepatic architecture. Focal areas of hepatocellular necrosis with inflammatory cells were frequently observed to infiltrate the hepatic parenchyma and the portal tract of hepatic lobules. Some hepatocytes showed vesicular nuclei, while others exhibited dark pyknotic nuclei accompanied by cytoplasmic vacuolization. The sinusoidal spaces displayed slight alterations. The detected hepatocellular vacuolation suggests fatty degeneration (steatosis) or hydropic changes induced by sertraline administration (Figure 3A–D). On the other side, sections from the livers of rats treated with both folic acid and sertraline displayed relatively preserved hepatic architecture with organized hepatic lobules and distinct central veins. The hepatocytes appear mostly normal, with intact cytoplasm and vesicular nuclei. Inflammatory infiltrates and vacuolations are minimal compared to sertraline-treated groups, and the sinusoids appear relatively normal with no significant inflammatory infiltration (Figure 3E–H). Overall, folic acid significantly improves histological architecture compared to sertraline-treated groups, suggesting that folic acid plays a protective role in reducing sertraline-induced hepatotoxicity.

**Figure 4:**
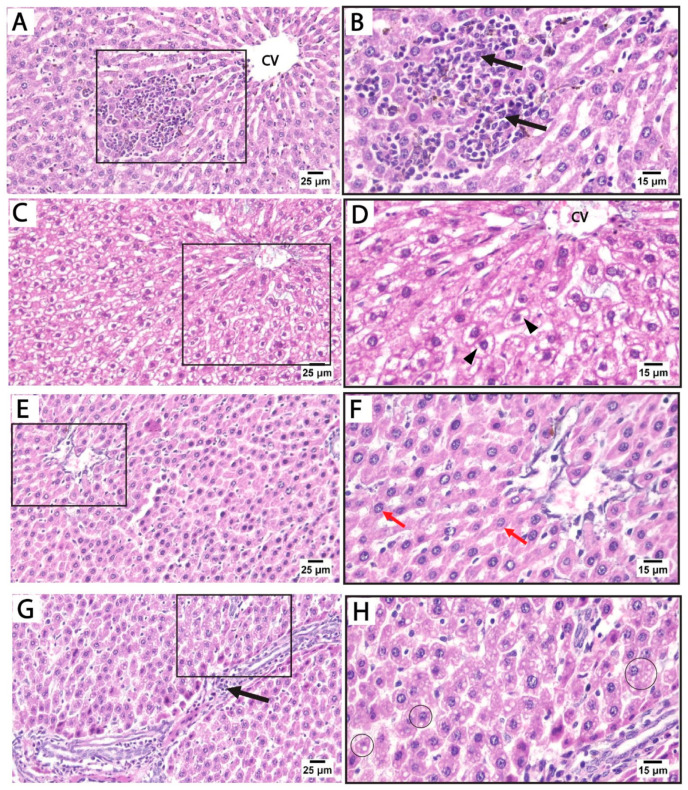
Photomicrograph of H&E-stained liver sections from treated rats euthanized immediately 14 days after giving birth. (**A**–**D**) treated with sertraline: (**A**,**B**) liver section showing massive inflammatory cell infiltration (black arrows) in the hepatic parenchyma around the central vein (CV). (**C**,**D**) Liver section showing disorganized hepatocyte cords near the congested central vein (CV). Vacuolated hepatocytes (arrowheads) are prominent. (**E**–**H**) treated with folic acid + sertraline: (**E**,**F**) Liver section showing normal cytoplasm with intact cytoplasm and vesicular nuclei (Red arrows). Hepatic sinusoids appear normal, with no significant evidence of inflammation or structural disruption. (**G**,**H**) Liver section displays hepatocytes with minimal vacuolation (circles). ((**A**,**C**,**E**,**G**): scale bar 25 μm), ((**B**,**D**,**F**,**H**): scale bar 15 μm), (**B**,**D**,**F**,**H**) are higher magnification of the areas marked by the square in (**A**,**C**,**E**,**G**), respectively.

Histological findings in pregnant rats 14 days postpartum: Light microscopy of H&E-stained liver sections from rats treated with sertraline and euthanized 14 days after birth revealed massive inflammatory cell infiltration in the hepatic parenchyma, particularly around the central vein. Some hepatocytes exhibited vacuolated cytoplasm with dark nuclei. Hepatocellular vacuolation was detected after sertraline administration for 14 postpartum, suggesting fatty degeneration or hydropic changes (Figure 4A–D). These findings highlight that the hepatotoxic effects of sertraline on the histological architecture of the liver are milder in rats euthanized 14 days postpartum when compared to more prominent damage observed in the sertraline-treated group euthanized immediately after birth. Conversely, sections from the livers of rats treated with folic acid and sertraline and euthanized 14 days postpartum demonstrate improved hepatic architecture compared to the sertraline-treated groups, indicating the protective effects of folic acid. Minimal vacuolation is observed, suggesting reduced cellular stress or damage. Hepatic sinusoids appear normal, with no significant evidence of inflammation or structural disruption (Figure 4E–H). These histological findings indicate that folic acid supplementation mitigates sertraline-induced hepatotoxicity.

Liver histopathology was assessed using Suzuki’s scoring system, evaluating sinusoidal congestion, hepatocellular vacuolation, and parenchymal necrosis (Table 3). In both control and folic acid-only groups at 0 h and 14 d, no histological abnormalities were observed, with scores of zero across all parameters, indicating preserved hepatic architecture. In contrast, rats treated with sertraline alone showed significant hepatic injury at both timepoints. At 0 h, sertraline induced marked sinusoidal congestion, vacuolation, and necrosis, all significantly higher than the respective control and folic acid groups (*p* < 0.05). These pathological changes persisted and were slightly more pronounced at 14 d, with mean scores of 2.5–2.62, demonstrating progressive liver damage.

Co-administration of folic acid with sertraline substantially attenuated the histopathological damage. At 0 h, the combination group showed significantly lower scores compared to the sertraline-treated group, particularly for necrosis (1.12 ± 0.64 vs. 2.75 ± 0.46). A similar protective effect was observed at 14 d, with moderate improvements in all three parameters, indicating the hepatoprotective effect of folic acid. Statistical comparisons revealed that these improvements were significant compared to the sertraline-treated group but remained significantly higher than that of the controls. These findings suggest that folic acid mitigates sertraline-induced liver injury, although partial damage persists, especially with prolonged exposure.

### 3.4. Immunohistochemical Analysis

**Figure 5:**
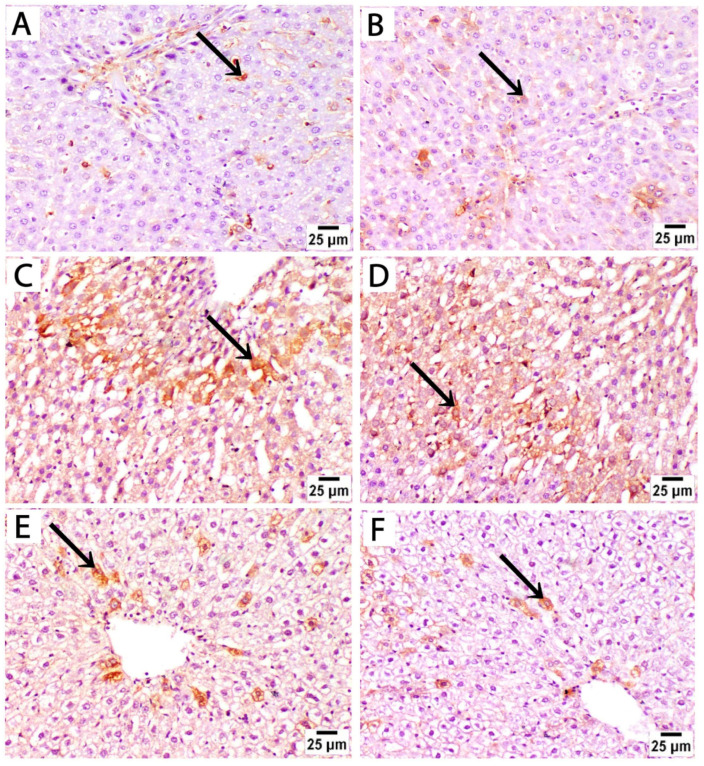
Photomicrographs of immunohistochemically stained liver sections by caspase 3 from treated rats euthanized immediately after birth. (**A**,**B**) The control group at both time points shows low expression of caspase 3, and positive brown cytoplasmic staining is sparsely localized in a few scattered hepatocytes (black arrow). (**C**,**D**) Liver sections from rats treated with sertraline show strong positive caspase 3 immunostaining in a large number of hepatocytes in broader areas of liver parenchyma. (**E**,**F**) Liver sections from rats treated with sertraline and folic acid show reduced intensity and extent of caspase 3 staining, as positive staining is confined to isolated hepatocytes near the central vein (caspase 3, scale bar 25 μm).

To assess the effect of sertraline and folic acid on apoptosis, caspase 3 expression was analyzed in liver sections from different experimental groups. Sections from controls at both time points showed low expression of caspase 3, as positive caspase 3 staining (brown) was sparsely localized in a few scattered hepatocytes, indicating no significant apoptotic activity beyond normal homeostasis (Figure 5A,B).

**Figure 6:**
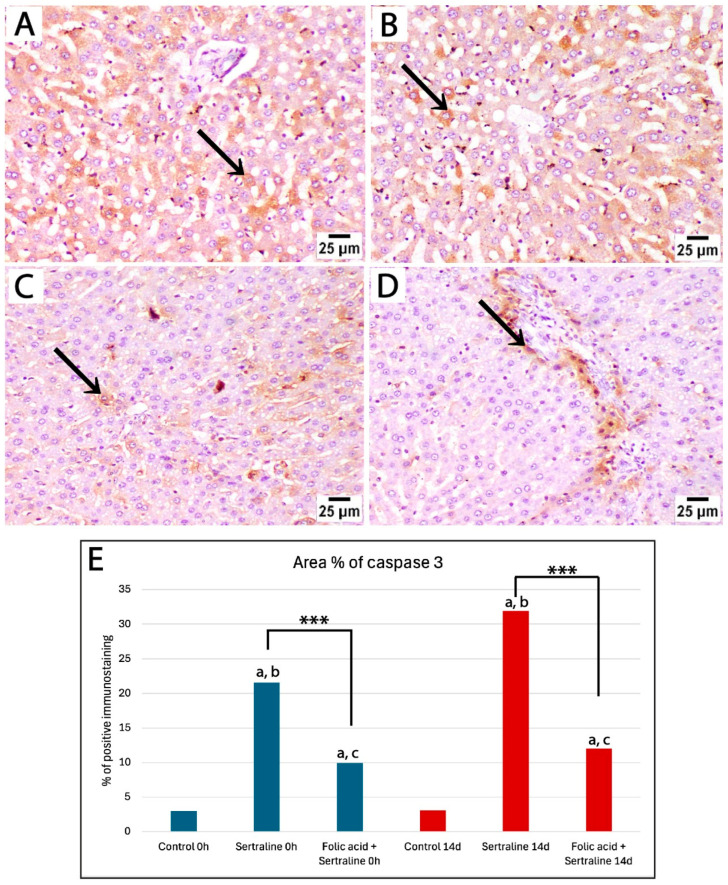
Photomicrographs of immunohistochemically stained liver sections by caspase 3 from treated rats euthanized 14 days after giving birth. (**A**,**B**) Liver sections from rats treated with sertraline show strong positive staining (black arrow) in a large number of hepatocytes in broader areas of liver parenchyma. (**C**,**D**) Liver sections from rats treated with sertraline and folic acid display weak caspase 3 expression, with positive staining (black arrow) confined to isolated hepatocytes or regions around the portal tract (caspase 3, scale bar 25 μm). (**E**) Bar chart showing the area percentage of positive immunostaining for caspase 3 in liver sections across different experimental groups. *** Significant difference between sertraline-treated and sertraline and folic acid groups; ^a^ significant difference from controls at the same time point; ^b^ significant difference between sertraline-treated groups at 0 h and 14 d; ^c^ significant difference between sertraline- and folic acid-treated groups at 0 h and 14 d.

Liver sections from rats treated with sertraline and euthanized immediately after giving birth showed a significant increase in caspase 3 expression compared to controls, as evidenced by increased area percentage of caspase 3 immunostaining (Figure 5E). Strong positive caspase 3 staining in a large number of hepatocytes in broader areas of liver parenchyma was observed. This detected pattern of high caspase 3 expression indicates increased apoptosis after sertraline administration (Figure 5C,D). Administration of both sertraline and folic acid followed by euthanasia immediately after birth was associated with a significant reduction in caspase 3 expression compared to the sertraline-treated group (Figure 5E,F) and (Figure 6E). The intensity and extent of caspase 3 staining were reduced, as positive staining was confined to isolated hepatocytes near the central vein. Of note, this group still showed significantly higher expression of caspase 3 compared to the controls (*p* < 0.001) (Figure 6E). This finding indicated that the hepatocytes exhibited minimal apoptotic activity, suggesting a protective effect of folic acid in mitigating sertraline-induced apoptosis.

Then, an assessment of the effects of sertraline and folic acids on caspase 3 expression in the livers of pregnant rats 14 days after giving birth was carried out. Liver sections from the sertraline-treated group exhibited widespread and intense positive staining, indicating significant apoptotic activity in hepatocytes (Figure 6A,B). The area percentage of caspase 3-positive immunostaining in the sertraline-treated group was significantly different from the control group (Figure 6E). In contrast, the sertraline + folic acid-treated group displayed reduced caspase 3 expression compared to the sertraline-treated group, with positive staining confined to isolated hepatocytes or regions around the portal tract (Figure 6C–E). This reduction suggests that folic acid mitigated sertraline-induced apoptosis, demonstrating its hepatoprotective effect.

Our morphometric analysis of the area percentage of caspase 3-positive immunostaining showed significantly high expression of caspase 3 in the sertraline-treated group at the time of giving birth and 14 days postpartum compared to controls. Notably, caspase 3 expression in the sertraline-treated 14 d group was significantly higher than that in the 0 h group (*p* < 0.001). The administration of folic acid with sertraline significantly reduced caspase 3 expression at 0 h and 14 d compared to the sertraline-treated groups (*p* < 0.001). In addition, there was a significant reduction in caspase 3 expression in the sertraline + folic acid-treated group euthanized immediately after giving birth compared to those assessed at 14 days postpartum (*p* = 0.03) (Figure 6E). Overall, these findings indicate that rats euthanized at 14 days postpartum are more likely to have higher hepatic stress and incidence of apoptosis.

## 4. Discussion

The current study provides crucial evidence that folic acid supplementation effectively mitigates sertraline-induced hepatotoxicity in pregnant albino rats. These protective effects are evidenced by the detected improvements immediately after giving birth and 14 days postpartum in biochemical parameters, histological architecture of hepatic tissue, and apoptotic activity. These findings have significant implications for pregnant women who require sertraline therapy for mood disorders, complementing its well-known role in preventing neural tube defects.

The results of the current study demonstrated that sertraline administration induced dyslipidemia, as a significant increase in total cholesterol (CHO), triglycerides (TGs), and low-density-lipoprotein cholesterol (LDL-c) levels was detected. Importantly, the rise in lipid parameters was more pronounced 14 days postpartum compared to the rats assessed immediately after giving birth, suggesting a cumulative metabolic burden of sertraline over time. These detected alterations in lipid profiles are consistent with findings in previous studies that associated the administration of selective serotonin reuptake inhibitors (SSRIs) with metabolic disturbances, potentially increasing the risk of nonalcoholic fatty liver disease [36]. Furthermore, this study demonstrated that sertraline administration during pregnancy was also associated with a significant increase in liver enzymes (AST and ALT), which indicates hepatocellular injury. The elevation in AST and ALT observed in the sertraline-treated groups was particularly distinct in postpartum rats (14 days after birth), suggesting that prolonged exposure to sertraline exacerbates hepatic stress. These results are in line with a previous study suggesting that chronic sertraline administration induced toxic effects on hepatic tissues by inducing oxidative stress, altering cytochrome P450 enzyme metabolism, and reducing gene expression of drug-metabolizing enzymes [6,7].

Liver sections from sertraline-treated rats showed disrupted hepatic architecture, hepatocellular vacuolization, and inflammatory infiltration, which are hallmarks of drug-induced liver injury, mainly in rats treated with sertraline for 14 days postpartum. Consistently with our histological findings, sertraline or its metabolites may trigger an immune response, leading to the infiltration of inflammatory cells, such as lymphocytes and eosinophils, into hepatic tissues [7,37]. This immune-mediated mechanism is supported by our results, where massive inflammatory cell infiltration in liver parenchyma was noted after sertraline administration for 14 days postpartum. Additionally, inflammatory processes associated with sertraline administration may result in hepatocyte death or necrosis, leading to hepatocellular injury and tissue repair processes that involve fibrosis if the injury is chronic.

The immune-modulatory effects of sertraline were proved recently by Önal et al. [38], who examined the influence of sertraline on the levels of cytokines (TNF-a, IL-6, IL-12p40, GM-CSF) in the macrophage cell line. Additionally, patients with major depression showed significantly elevated levels of proinflammatory cytokines (TNF-a, IL-2, IL-12) after sertraline use [39]. The cytoplasmic vacuolization of hepatocytes observed in our histological analysis can be explained by the findings of De Boer and Sherkerde [40]**,** who demonstrated that sertraline can impair lipid metabolism and induce lipid accumulation in hepatocytes, leading to vacuolization. Furthermore, SSRIs, including sertraline, may impair mitochondrial function, leading to cellular energy depletion and vacuolization [41].

In the present study, the sertraline-treated group demonstrated widespread and intense positive caspase 3 immunostaining, indicating significant apoptotic activity in hepatocytes. This is consistent with previous findings that link sertraline-induced oxidative stress with apoptosis-mediated liver damage [42,43]. Similarly, the detected increase in caspase 3 expression aligns with the findings of Then et al. [44], who reported that sertraline induces mitochondrial dysfunction, reactive oxygen species (ROS) generation, and apoptosis. These hepatotoxic effects were associated with increased intracellular calcium levels and activation of caspase 3 and poly(ADP-ribose) polymerase (PARP), indicating the involvement of the intrinsic apoptotic pathway.

The coadministration of folic acid with sertraline significantly mitigated sertraline-induced dyslipidemia by significantly lowering CHO and LDL-c levels. This effect is in accordance with previous reports that demonstrated folic acid’s ability to modulate lipid metabolism and reduce hyperlipidemia risk [8,45]. Also, Yang et al. demonstrated that folic acid helps reduce hyperlipidemia and its complications, as it lowers triglyceride levels while increasing HDL-c, supporting overall lipid metabolism [12]. Another study reported that folic acid affects measured lipid metabolism by improving and regulating lipid profile metabolism, especially in high-dose supplementations (>1 mg/day) [46]. The mechanism underlying these protective effects can be attributed to the role of folic acid in reducing oxidative stress, reducing hepatic fat accumulation, regulating lipid metabolism, and enhancing the liver’s ability to process fats [8,45,47]. Moreover, folic acid attenuates high-fat diet-induced steatohepatitis in rats by restoring peroxisome proliferator-activated receptor α (PPARα) levels via a sirtuin 1 (SIRT1)-dependent pathway, improving hepatic lipid metabolism and reducing inflammation [48].

Similarly, folic acid supplementation significantly improved liver function markers, reducing AST and ALT levels closer to the control group, suggesting that folic acid alleviates hepatic stress. This is consistent with two previous studies that demonstrated the protective effects of folic acid against isoniazid and alcohol-induced liver injury in mice [49,50]. These protective effects can be explained by the ability of folic acid to restore gut microbiota balance and reduce intestinal inflammation. This modulation leads to decreased lipopolysaccharide leakage, thereby inhibiting the Toll-like receptor 4/nuclear factor kappa B (TLR4/NF-κB) signaling pathway and reducing liver injury [50]. Additionally, folic acid plays a crucial role in homocysteine metabolism, supporting DNA methylation processes that are essential for cellular repair and reducing oxidative damage [8,12].

Histological examination demonstrated that the folic acid-treated group’s structural disruption of hepatic architecture was significantly attenuated. Hepatocyte organization was largely preserved, and inflammatory cell infiltration was minimal. These findings are consistent with a previous study that reported that folic acid supplementation was associated with ameliorated hepatic lipid accumulation and inflammatory infiltrate [11]. Notably, folic acid supplementation significantly reduced caspase 3 expression, suggesting its role in mitigating apoptosis through antioxidant and anti-inflammatory mechanisms. These results are consistent with previous studies demonstrating folic acid’s ability to attenuate oxidative stress-induced apoptosis in hepatic cells [46].

These results support the conclusion that folic acid supplementation may have additional effects beyond the previously well-known role of preventing fetal neural tube defects during pregnancy. Considering the frequent use of sertraline in pregnant women who have mood changes, the potential hepatotoxicity of this drug needs additional clinical exploration, particularly with prolonged administration. However, while this study provides valuable insights into the hepatoprotective effects of folic acid, further research is needed to confirm these findings in human populations. Future studies should focus on dose optimization, the duration of folic acid supplementation, and its long-term effects on maternal and fetal health.

### Study Limitations and Future Perspectives

This study has several limitations that should be acknowledged. Firstly, the absence of a postpartum recovery-only group limited our ability to distinguish the specific effects of folic acid supplementation from the natural physiological recovery that may occur during the postpartum period. While the improvements observed are encouraging, it remains possible that folic acid facilitated or enhanced an ongoing recovery process rather than acting as the sole therapeutic factor. Secondly, the study lacks mechanistic validation: although improvements in biochemical and histopathological outcomes were noted, we did not explore specific molecular pathways such as antioxidant gene expression, inflammatory signaling, or apoptotic cascades. Using the albino rats as a model for pregnancy might not be the best replicate for human pregnancy, especially for the duration of pregnancy and hormonal levels. Another limitation was that the study focused on the short-term analysis of a single dose of folic acid daily during the pregnancy and postpartum periods. Assessing different doses or longer supplementation periods could lead to varying results. Finally, only two tests of liver function were measured in the current study. Other liver functions, such as prothrombin time, are important to investigate in pregnancy. Therefore, we recommend using such tests in further studies.

## 5. Conclusions

In conclusion, this study demonstrates that sertraline administration during pregnancy induces significant hepatotoxic effects, including dyslipidemia, elevated liver enzymes, histological aberrations, and increased apoptosis. Folic acid supplementation can effectively alleviate these adverse effects, as shown by improving lipid metabolism, restoring liver function, improving the histological architecture of the liver, and reducing apoptotic activity. While these findings highlight the potential protective role of folic acid during sertraline therapy in pregnancy, it is important to note that the results are based on an animal model. Therefore, extrapolation to human clinical scenarios should be approached with caution, and further research in human populations is needed to confirm these observations.

## Figures and Tables

**Table 1:** Means and standard deviations of CHO, TG, HDL-c, and LDL-c in different groups.

Variable	CHO (mg/dL)	TG (mg/dL)	HDL-c (mg/dL)	LDL-c (mg/dL)
Group				
Control 0 h	89.18 ± 4.1	80.91 ± 2.5	47.2 ± 1.6	46.3 ± 3.3
Folic acid 0 h	91.45 ± 6.2	80.16 ± 2.97	46 ± 1.6	47.1 ± 4.86
Sertraline 0 h	162.53 ± 9.78 ^a,b^	147.7 ± 10.8 ^a,b^	45.3 ± 2.4	78.65 ± 3.25 ^a,b^
Folic acid + sertraline 0 h	99.26 ± 3.9 ^a,b,c^	97.90 ± 3.89 ^a,b,c^	46.09 ± 2.73	48.75 ± 1.61 ^c^
Control 14 d	88.6 ± 4.74	80.41 ± 5.08	47.0 ± 2.98	44.05 ± 2.74
Folic acid 14 d	91.65 ± 6.05	80.66 ± 1.87	46.787 ± 2.82	44.52 ± 3.89
Sertraline 14 d	220.05 ± 13.4 ^a,b,c,d,e,f^	177.4 ± 12.4 ^a,b,c,d,e,f^	45.58 ± 3.8	96.88 ± 4.8 ^a,b,c,d,e,f^
Folic acid + sertraline 14 d	121.59 ± 9.54 ^a,b,c,d,e,f,g^	116.1 ± 6.2 ^a,b,c,d,e,f,g^	48.75 ± 1.612	72.46 ± 3.4 ^a,b,c,d,e,f,g^
F	363.0108	295.55	1.895	272.9104
*p*-value	0.000	0.000	0.082	0.000

One-way ANOVA followed by Tukey’s post hoc test to investigate differences in total cholesterol (CHO), triacylglycerol (TG), high-density lipoprotein (HDL), and low-density lipoprotein (LDL) in groups 1–8, Values were deemed statistically significant when *p* < 0.05. ^a^: statistically significant difference from control 0 h; ^b^: statistically significant difference from folic acid 0 h; ^c^: statistically significant difference from sertraline 0 h; ^d^: statistically significant difference from folic acid + sertraline 0 h; ^e^: statistically significant difference from control 14 d; ^f^: statistically significant difference from folic acid 14 d; ^g^: statistically significant difference from sertraline 14 d.

**Table 2:** Means and standard deviations of ALT and AST in different groups.

Variable	Alanine Aminotransferase (ALT)	Aspartate Aminotransferase (AST)
Group		
Control 0 h	13.4 ± 1.21	21.12 ± 1.86
Folic acid 0 h	14.03 ± 0.72	21.3 ± 1.2
Sertraline 0 h	49.03 ± 2.09 ^a,b^	45.3 ± 3.1 ^a,b^
Folic acid + sertraline 0 h	25.96 ± 1.73 ^a,b,c^	28.40 ± 2.30 ^a,b,c^
Control 14 d	13.7 ± 1.45	21.49 ± 1.02
Folic acid 14 d	14.81 ± 1.23	21.87 ± 2.0
Sertraline 14 d	70.1 ± 1.8 ^a,b,c,d,e,f^	66.47 ± 3.52 ^a,b,c,d,e,f^
Folic acid + sertraline 14 d	36.31 ± 2.66 ^a,b,c,d,e,f,g^	34.40 ± 2.99 ^a,b,c,d,e,f,g^
F	1487.037	444.3912
*p*-value	0.000	0.000

One-way ANOVA was followed by Tukey’s post hoc test to investigate differences in ALT and AST in groups 1–8. Values are deemed statistically significant when *p* < 0.05. ^a^: statistically significant difference from control 0 h; ^b^: statistically significant difference from folic acid 0 h; ^c^: statistically significant difference from sertraline 0 h; ^d^: statistically significant difference from folic acid + sertraline 0 h; ^e^: statistically significant difference from control 14 d; ^f^: statistically significant difference from folic acid 14 d; ^g^: statistically significant difference from sertraline 14 d.

**Table 3:** Semiquantitative histopathological scoring of liver tissue in different experimental groups at 0 h and 14 days postpartum.

Variable	Sinusoidal Congestion	Vacuolation	Necrosis
Control 0 h	0.0 ± 0.0 ^c,d^	0.0 ± 0.0 ^c,d^	0.0 ± 0.0 ^c,d^
Folic acid 0 h	0.0 ± 0.0 ^c,d^	0.0 ± 0.0 ^c,d^	0.0 ± 0.0 ^c,d^
Sertraline 0 h	2.0 ± 0.76 ^a,b,d^	2.0 ± 0.76 ^a,b^	2.75 ± 0.46 ^a,b,d^
Folic acid + sertraline 0 h	1.12 ± 0.64 ^a,b,c^	1.75 ± 0.71 ^a,b^	1.12 ± 0.64 ^a,b,c^
Control 14 d	0.0 ± 0.0 ^g,h^	0.0 ± 0.0 ^g,h^	0.0 ± 0.0 ^g,h^
Folic acid 14 d	0.0 ± 0.0 ^g,h^	0.0 ± 0.0 ^g,h^	0.0 ± 0.0 ^g,h^
Sertraline 14 d	2.5 ± 0.53 ^e,f^	2.62 ± 0.52 ^e,f^	2.62 ± 0.52 ^e,f,h^
Folic acid + sertraline 14 d	1.62 ± 0.74 ^e,f^	1.75 ± 0.71 ^e,f^	1.75 ± 0.71 ^e,f,g^

Values are expressed as mean ± SD (n = 10). Superscript letters indicate statistically significant differences between groups (*p* < 0.05). ^a^: statistically significant difference from control 0 h; ^b^: statistically significant difference from folic acid 0 h; ^c^: statistically significant difference from sertraline 0 h; ^d^: statistically significant difference from folic acid + sertraline 0 h; ^e^: statistically significant difference from control 14 d; ^f^: statistically significant difference from folic acid 14 d; ^g^: statistically significant difference from sertraline 14 d; ^h^: statistically significant difference from folic acid + sertraline 14 d.

## Data Availability

All data supporting the findings of this study are available upon reasonable request.
